# Dupilumab as an Effective Adjuvant Therapy for Corticosteroid‐Refractory Lichenoid‐Bullous Dermatitis Secondary to Pembrolizumab

**DOI:** 10.1111/ajd.70147

**Published:** 2026-05-29

**Authors:** Brandon Tan, Gaurav N. Pathak, Alexis Lara Rivero

**Affiliations:** ^1^ Department of Dermatology St. George Hospital Sydney New South Wales Australia; ^2^ Faculty of Medicine University of New South Wales Sydney New South Wales Australia; ^3^ Department of Dermatology Rutgers Robert Wood Johnson Medical School Somerset New Jersey USA

**Keywords:** breast cancer, dupilumab, immune‐checkpoint inhibitors, immunotherapy, lichenoid drug reaction, pembrolizumab

Dear Editor,

Immune checkpoint inhibitors (ICIs) have transformed the therapeutic landscape in oncology; however, cutaneous immune‐related adverse events (cirAEs), affecting 30%–50% of patients [1], often complicate management. Lichenoid eruptions occur in approximately 6% of cirAEs and may lead to interruption or discontinuation of immunotherapy, potentially impacting prognosis. Current guidelines do not specifically address lichenoid reactions, limiting treatment to systemic corticosteroids and discontinuation of immunotherapy [2]. Dupilumab, an interleukin‐4 receptor alpha (IL‐4Rα) antagonist targeting IL‐4/IL‐13 signalling, has recently garnered attention as potential targeted therapy for ICI‐induced lichenoid dermatitis (ICILD), based on RNA in situ hybridisation studies demonstrating IL‐13 expression in affected skin [3]. We present a unique case of severe lichenoid‐bullous reaction successfully treated with dupilumab, without clinical evidence of progression.

A 61‐year‐old woman was admitted to hospital for urgent dermatological review following a 10‐day history of widespread confluent erythematous rash with tense bullae (Figure 1A), oral mucositis (Figure 1B), and constitutional symptoms. She had received combination gemcitabine, carboplatin, and pembrolizumab nine months earlier for metastatic triple‐negative PD‐L1‐positive breast cancer, followed by two doses of maintenance pembrolizumab monotherapy within the past month. Past medical history included type 1 diabetes mellitus and pulmonary embolism. The patient was commenced on silver‐based wound dressings, topical betamethasone dipropionate 0.05% ointment, oral prednisolone 50 mg twice daily, and oral mycophenolate mofetil (MMF) 1000 mg twice daily. By week 2, progressive mucosal involvement and rapid denuding of palmoplantar skin (Figure 1C,D) were observed. Skin biopsy demonstrated moderate‐to‐severe interface dermatitis, subepidermal clefting, and numerous Civatte bodies without epidermal necrosis (Figure 2). Direct immunofluorescence and autoimmune blistering serology were negative. Given involvement of greater than 50% body surface area and oral mucosa, the diagnosis of grade 4 lichenoid‐bullous drug reaction was made, warranting permanent discontinuation of pembrolizumab.

**FIGURE 1:**
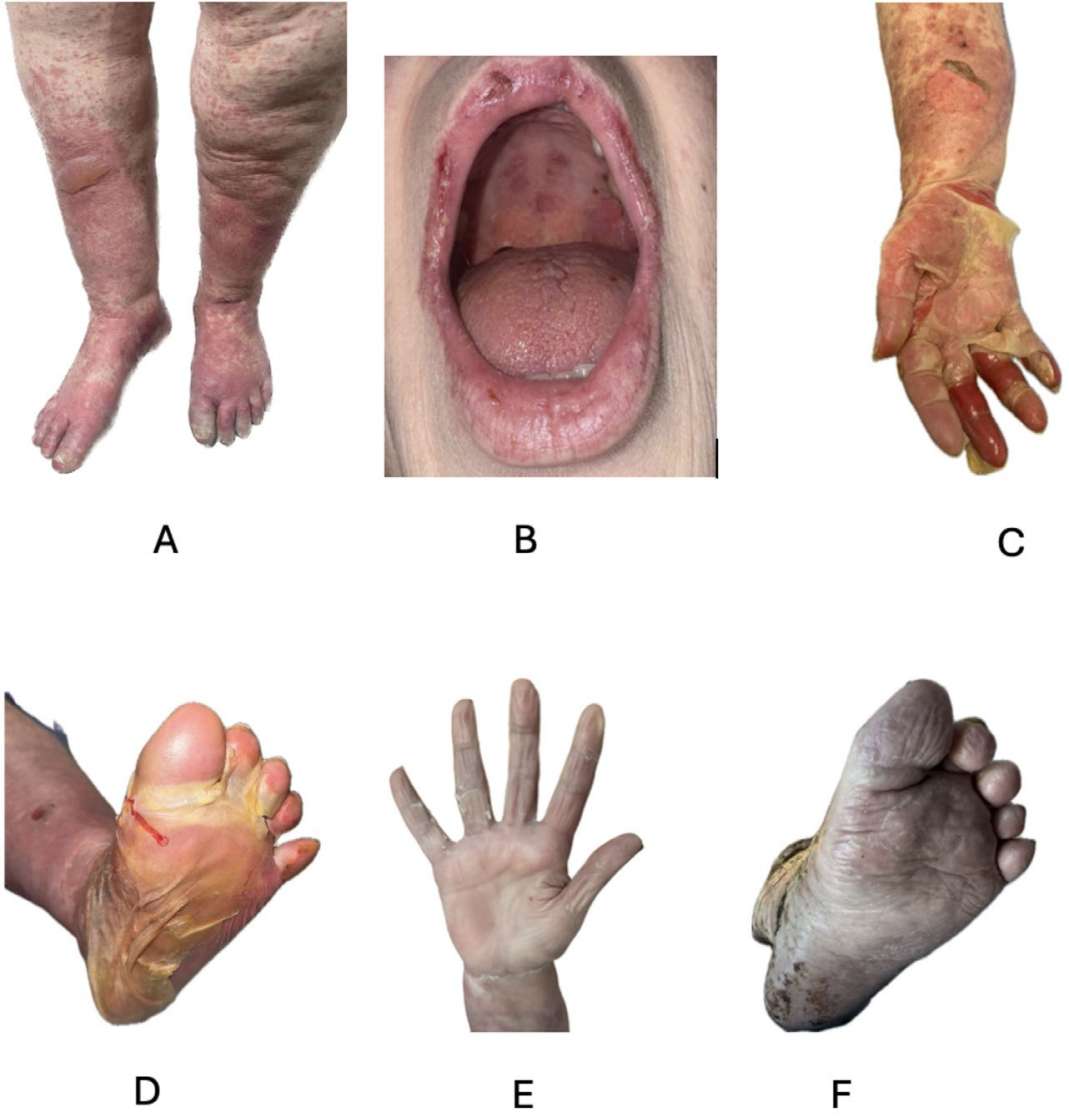
Clinical images demonstrating confluent erythematous eruption more prominent in distal extremities with tense bullae formed (A), oromucosal erosions (B), complete denuding of palmoplantar skin in early course of disease (C‐D); complete re‐epithelialisation of palmoplantar skin within first two weeks of dupilumab commencement (E‐F).

**FIGURE 2:**
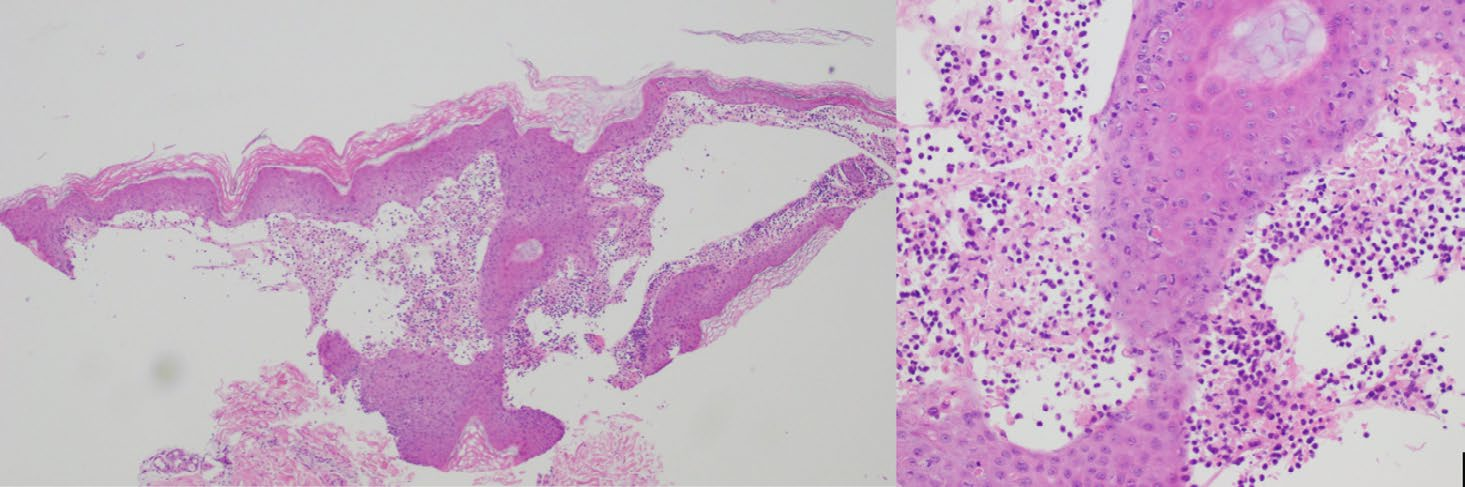
Skin biopsy on haematoxylin and eosin staining demonstrating an attenuated epidermis without evidence of necrosis (left), moderate‐to‐severe interface dermatitis with subepidermal clefting and numerous Civatte bodies (right).

In week 3, wound healing remained slow with persistent painful oromucosal erosions. Intravenous rituximab 1000 mg was administered and prednisolone was slowly tapered. The admission was subsequently complicated by septic shock secondary to *Pseudomonas aeruginosa* bacteraemia in the context of profound immunosuppression, steroid‐induced hyperglycaemia and delayed wound healing, requiring brief intensive care unit (ICU) admission for vasopressor support. In week 5, off‐label subcutaneous dupilumab was commenced (600 mg loading dose, then 300 mg fortnightly), along with prednisolone 50 mg daily and same doses of MMF and topical corticosteroids. After two doses of dupilumab, no new lesions were observed and complete palmoplantar re‐epithelialisation was achieved (Figure 1E,F). Prednisolone was tapered and ceased by week 10. The patient was discharged in week 13 on dupilumab, MMF and topical treatments, with sustained improvement in wound healing, pain, and glycaemic control noted at follow‐up in week 16.

**Figure:**
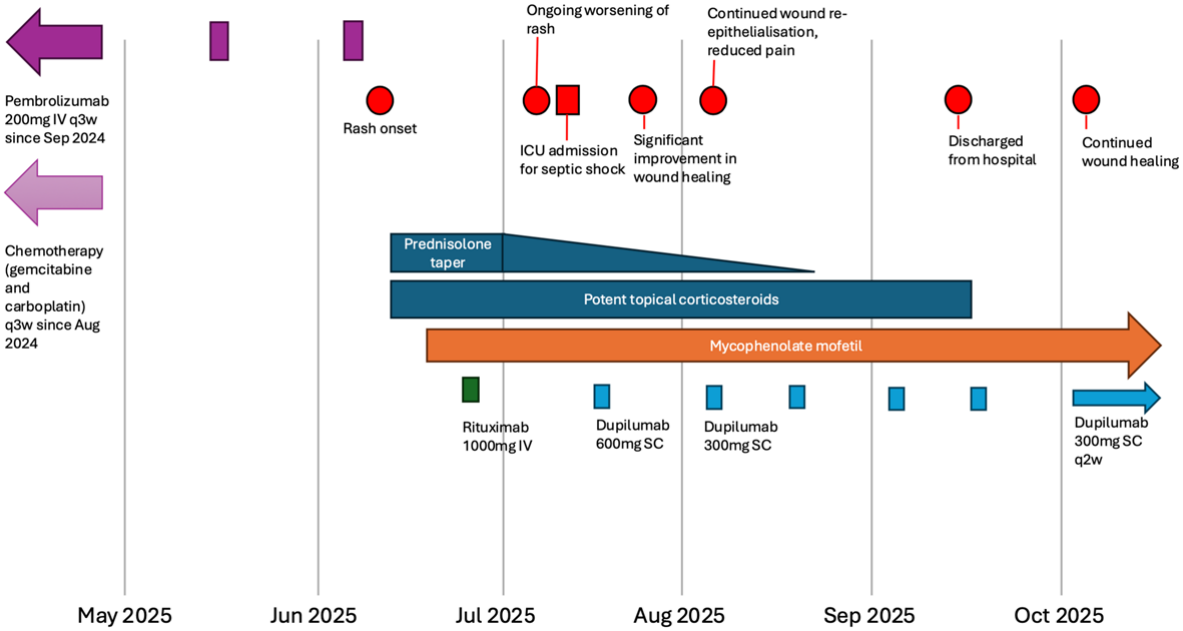
GRAPHIC | Timeline of events demonstrating latency period to onset of cutaneous drug eruption, duration of treatments used and time to clinical response.

This case is among the first to support an emerging role for dupilumab in treating ICILD. By targeting IL‐4/IL‐13 signalling and Th2‐driven inflammation [3], dupilumab may reduce reliance on high‐dose corticosteroids and associated metabolic adverse effects. Recent studies support clinical efficacy and safety of dupilumab in treating classic lichen planus [4] and bullous pemphigoid [5], highlighting its role in ICILD and immunobullous cirAEs, respectively [3]. Future studies should evaluate dupilumab on a larger scale to establish its safety and efficacy in treating severe cirAEs.

## Funding

The authors have nothing to report.

## Consent

Written consent for publication of clinical information and photographs was obtained from the patient prior to article submission.

## Conflicts of Interest

The authors declare no conflicts of interest.

## Data Availability

The data that support the findings of this study are available from the corresponding author upon reasonable request.
